# Determination of Saffron Volatiles by HS-SBSE-GC in Flavored Cured Ham

**DOI:** 10.3390/molecules26072073

**Published:** 2021-04-04

**Authors:** Elena M. Gómez-Sáez, Natalia Moratalla-López, Cándida Lorenzo, Herminia Vergara, Gonzalo L. Alonso

**Affiliations:** 1 Benibaldo, S.A.U., 02007 Albacete, Spain; Calidad@benibaldo.com; 2 Cátedra de Química Agrícola, ETSI Agrónomos y de Montes, Universidad de Castilla-La Mancha, Campus Universitario, 02071 Albacete, Spain; Natalia.Moratalla@uclm.es (N.M.-L.); Candida.Lorenzo@uclm.es (C.L.); 3 Regional Development Institute, Food Quality Section, Universidad de Castilla-La Mancha, 02071 Albacete, Spain; Herminia.Vergara@uclm.es

**Keywords:** ham, flavoring, safranal, *Crocus sativus* L., HS-SBSE-GC/MS

## Abstract

At present, the development of new agri-food products, including flavored meat products presented in ready-to-eat vacuum packs, is encouraged. The addition of ingredients used as flavoring agents creates the need to be able to determine the volatile compounds responsible for their characteristic aroma. The aim of this study is to propose, develop, and validate a new method that uses headspace-stir bar sorptive extraction-gas chromatography/mass spectrometry (HS-SBSE-GC/MS) to determine the saffron aroma in cured ham flavored with this spice. Results showed that safranal was the main volatile compound that could be identified and quantified in cured ham flavored with saffron. This analytical method was adequate in terms of linearity, selectivity, sensitivity, and accuracy. To our knowledge, this is the first time that an HS-SBSE-GC/MS method for determining the saffron aroma of flavored cured ham has been developed and validated, and it is of interest to agri-food industries.

## 1. Introduction

One of the cured meat products most valued by Mediterranean consumers is ham. The quality of this product varies according to the pig breed and diet and the processing of the meat. Thus, different hams can be distinguished on the basis of pig breed, for example, Iberian and White pig hams [1]; feeding method, for example, in a corn-based pasture or programmed feeding where the animal is housed [2]; or the application of different processing techniques [3,4]. Furthermore, ham quality is determined by its physicochemical (pH, color) and sensory characteristics (mainly its aroma and flavor, which are influenced by the type of cut or presentation). In Spain, there are professional “ham cutters” because the precision of slicing influences greater or lesser acceptance by consumers. The change in lifestyle that has been underway for a few years means that ham cutting cannot be performed by the consumers themselves and that ready-to-eat slices of cured ham are sold in vacuum packages [5]. This implies the addition of a new phase in the entire industrial process for obtaining the food product. The slicing–packaging process also affects the quality of the final product and offers new possibilities to influence the flavor and aroma of the ham. The generation of aromas can take place inside the meat itself, which is typical of the traditional curing process [6], or by exogenous pig back fat incorporation (absorption) [7]. With this new process (slicing–packaging), spices or other products can be added to enhance sensory characteristics by adsorption of suitable volatiles.

Saffron is a highly valued spice that imparts color, flavor, and aroma to food. The compounds responsible for these properties are a group of carotenoids, crocetin sugar esters, picrocrocin, and a wide array of ketones and terpenic aldehydes, with safranal being the most important compound [8]. Picrocrocin is the most important substance responsible for the characteristic bitter taste of saffron [9]. In addition, picrocrocin is associated with the aroma of saffron because it is considered a safranal precursor generated during dehydration [10]. More than 100 volatile compounds have been detected in the aroma of saffron; safranal contributes to more than 70% of the aroma in Spanish saffron, followed by isophorone, 2,6,6-trimethyl-2-cyclohexene-1,4-dione (tMCHdO), and linalool [11]. Different spices or herbs are added to food to transmit a special aroma or extend the life or enhance the flavor of the final product [12]. Saffron has already been used as an ingredient in Spanish sheep milk pressed cheese to study this effect [13]. Sensory analysis suggests that the differences in the flavor of the cheese with different concentrations of saffron were less evident as the ripening period increased [14], and the different concentrations of saffron used could be determined in the volatile fraction of these cheeses [15].

To our knowledge, no analytical method has so far been developed to assess the transfer of saffron, in terms of its aroma, or any other spice, to cured ham. The most suitable analytical technique to determine the transfer in flavor is gas chromatography (GC) using the headspace (HS) technique because extraction with liquid solvents and subsequent injection would extract the apolar compounds from the ham, which could interfere with the chromatographic resolution. Stir bar sorptive extraction (SBSE), which uses an absorbent polydimethylsiloxane (PDMS)-coated bar, has been successfully introduced and applied to analyze volatile components in different matrices such as breast milk [16] or to determine the key aromatic compounds of Longjing tea [17,18]. In addition, PDMS was successfully used as an absorbent in the HS technique in the analysis of volatile compounds of ham [19] and of saffron [20,21]. In both studies, solid-phase microextraction (SPME) was used. However, the stir bar had a greater absorption capacity than the fiber used in SPME because it has a greater volume of PDMS. Licón et al. [14] optimized an SBSE method to determine volatile compounds in sheep milk pressed cheese. This technique requires easy sample preparation, is rapid, and allows series of extractions simultaneously. The objective of the present study is to propose, develop, and validate a method based on HS-SBSE-GC to determine the volatile compounds of saffron in cured ham flavored with the spice.

## 2. Results and Discussion

### 2.1. Conditions for Extraction of Volatile Compounds from Saffron

**Figure 1:**
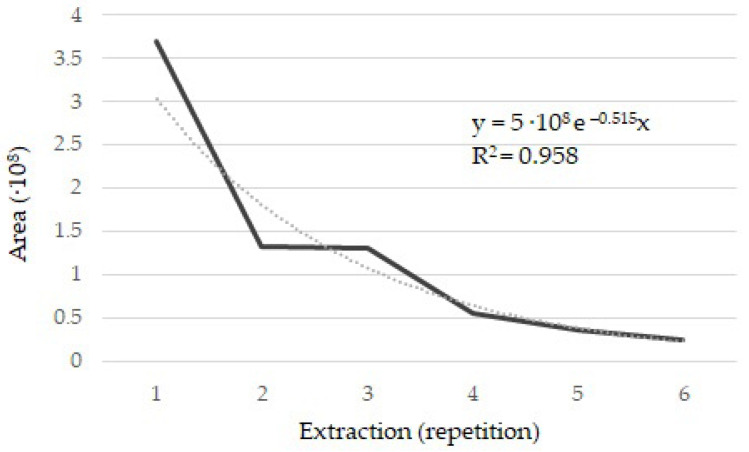
Progression of safranal peak area in saffron samples (33.5 mg powdered saffron) subjected to 30 °C for 40 min and purified using stir bar sorptive extraction in six consecutive extractions.

Prior to the analysis of volatile compounds, it was necessary to establish certain parameters to identify the optimal extraction conditions. Thus, the different factors studied were optimized sequentially. First, the quantity of powdered saffron and vial volume were considered. Previous research suggested that a quantity of saffron greater than 20 mg but not exceeding 40 mg could be used [15,20,21,22,23]. Therefore, 30 mg and 40 mg were the quantities under study. In addition, the saffron quantity used had to be consistent with the quantity added to flavor-cured ham; therefore, 33.5 mg was the quantity of powdered saffron chosen. Regarding vial volume, vials with the largest volume were selected, on the basis of the previous literature (10–20 mL), to leave as much free volume as possible for the volatile compounds obtained. In the second step, the retention temperature and heating time of extraction were studied. For this purpose, two different temperatures (oven at 30 °C and 45 °C) and six different heating times of extraction (10, 20, 30, 40, 50, and 60 min) were assessed. To evaluate these extraction conditions, three vials were used for each pair of conditions to be studied (36 vials in total). Thus, 33.5 mg of ground saffron was introduced into a 20 mL vial, the insert and the Twister stir bar were placed into the vial, and after its closure, it was subjected to the extraction conditions under study. From the data obtained, 30 °C and 40 min were determined to be optimal. An increase in temperature from 30 °C to 45 °C did not result in a higher content of saffron volatile compounds, and it could generate volatile compounds from the cured ham. Regarding heating time, an increase from 10 min to 40, 50, or 60 min led to a higher quantity of extracted compounds, with no differences among the last three times studied; thus, the shortest time among them was chosen. Finally, the extraction performance was evaluated under the established extraction conditions. Six extractions from the same vial were performed, and the saffron aroma was analyzed according to the present method. To assess this parameter, safranal peak area was used because this compound constitutes at least 70% of the volatile fraction of saffron [11]. Figure 1 shows the progression of the peak area of safranal over six extractions performed. The first extraction yielded 49.1% of the total safranal extracted in six consecutive extractions from the same vial.

### 2.2. Internal Method Validation

The linearity of the proposed method using the calibration graphs of the saffron components (safranal, isophorone, linalool, and 2,6,6-trimethyl-2-cyclohexene-1,4-dione) is shown in Table 1. 

The linear range of the calibration curves was assessed by calculating the regression line with the least-squares method [24]. Six concentrations were tested in duplicate, and regression lines were calculated for each compound. The correlation coefficients obtained ranged from 0.985 (2,6,6-trimethyl-2-cyclohexene-1,4-dione) to 0.999 (safranal), which indicated good linearity. The selectivity was assessed using the majority ion of the corresponding mass spectra for quantification, comparing the chromatograms obtained from the analyzed solutions. The proposed method showed good selectivity because the retention times of the standards and the volatile compounds of saffron obtained did not differ significantly (relative standard deviation (RSD) < 0.011%). 

The sensitivity and precision of the experimental procedure were also evaluated (Table 2). 

The sensitivity was determined on the basis of the LOD and LOQ of each standard used. The LOD and LOQ values ranged from 3.992 × 10^−4^ µg/20 mL vial (safranal) to 2.590 × 10^−2^ µg/20 mL vial (linalool) and from 1.210 × 10^−3^ µg/20 mL vial (safranal) to 7.847 × 10^−2^ µg/20 mL vial (linalool), respectively. These data suggested a high capacity for the identification and quantification of the saffron compounds under study.

The accuracy of the method was evaluated for each standard used by means of the %RSD values obtained for repeatability and reproducibility (intermediate precision) (Table 2). Both the repeatability and reproducibility results showed %RSD values lower than 4.05% for all compounds studied, highlighting the good accuracy of this analytical method according to the guidelines employed (RSD < 20%) [24]. 

Therefore, according to the results obtained for linearity, sensitivity, and accuracy (method performance characteristics), the proposed HS-SBSE-GC/MS method was successfully validated. 

### 2.3. Determination of Saffron Aroma in Cured Ham

To test the applicability of the present method, filleted cured ham flavored with saffron was analyzed. For this purpose, three samples each of three different ratios of powdered saffron to cured ham were analyzed using the HS-SBSE-GC/MS method. Approximately 200 mg of cured ham was placed into a 20 mL vial. The quantity of ham chosen was the largest quantity possible without interfering with the headspace of the vial, which is necessary for adsorption, and was selected considering the quantity of 33.5 mg established in the extraction conditions previously assessed. All analyses were performed in triplicate, with two measurements taken for each replicate. 

**Figure 2:**
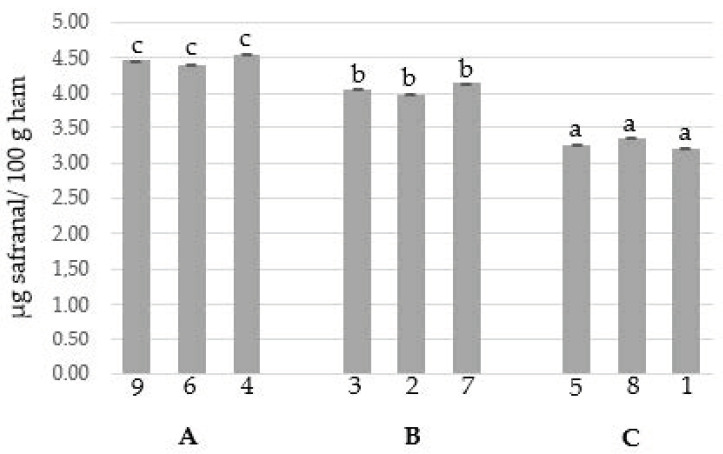
Mean values (3 × 2*n*) and standard deviation of safranal content of flavored cured ham samples (1–9) with different saffron ratios (**A**: high, **B**: medium, and **C**: low).

The HS-SBSE-GC/MS method developed and validated in this study was used to examine the volatile compounds of saffron in flavored cured ham. The saffron volatile compounds of the samples were quantified on the basis of the calibration curves generated in this study (Table 1). The analyzed samples reached values below their limit of quantification (LOQ) for some of the evaluated compounds, but all samples showed the presence of safranal. Therefore, safranal was the main compound which could be used to distinguish and classify cured ham flavored with saffron. The differences in aroma (safranal) among cured ham samples with different ratios of powdered saffron to cured ham were determined using ANOVA (*p* < 0.05) (Figure 2).

As seen in Figure 2, the analyzed flavored cured ham samples (1–9) showed significant differences (a–c) in safranal content (µg safranal/100 g flavored cured ham), which distinguished between samples belonging to a high (A), medium (B), or low (C) powdered saffron to cured ham ratio. Therefore, the HS-SBSE-GC/MS method fulfilled the purpose for which it was proposed, developed, and validated.

## 3. Materials and Methods

### 3.1. Samples and Reagents

*Samples*. Saffron from the Protected Designation of Origin (PDO) “Azafrán de La Mancha” was directly purchased from the producer Agrícola Técnica de Manipulación y Comercialización (Minaya, Albacete, Spain). Category I saffron was ground and characterized according to ISO 3632:2011 [25] ($A_{1\mathrm{cm}}^{1\%}$ 440 nm: 235 ± 2, $A_{1\mathrm{cm}}^{1\%}$ 257 nm: 97 ± 3, and $A_{1\mathrm{cm}}^{1\%}$ 330 nm: 22 ± 1). Cured ham was obtained from the company Benibaldo, S.A.U. (Albacete, Spain), and rice flour obtained from Panificadora Conquense Agrícola, S.A. (Cuenca, Spain), a usual product in the ham industry, was used in this study as a solid support to retain the patterns and for solid dilution of saffron. 

*Patterns*. A total of four patterns of the major aromatic components of saffron were used. Safranal (purity: 90%), linalool (purity: 97%), isophorone (purity: 97%), and 2,6,6-trimethyl-2-cyclohexene-1,4-dione (purity: 98%) were obtained from Sigma-Aldrich (Madrid, Spain).

*Solvent*. Cyclohexane of analytical grade was obtained from Panreac (Barcelona, Spain).

### 3.2. Selection of Conditions for Extraction of Volatile Compounds from Saffron

Volatile compound extraction was performed by the headspace-stir bar sorptive extraction (HS-SBSE) technique. For this purpose, a PDMS-coated stir bar (0.5 mm film thickness × 20 mm length; Twister, Gersterl GmbH, Mülheim an der Ruhr, Germany) was placed into the insert, and headspace vials were sealed with an aluminum crimp cap. The following variables were studied: quantity of powdered saffron used (30 and 40 mg), vial volume (10 and 20 mL), retention temperature (30 °C and 45 °C), and heating time (10, 20, 30, 40, 50, and 60 min).

### 3.3. Analysis of Volatile Compounds by Headspace-Stir Bar Sorptive Extraction-Gas Chromatography/Mass Spectrometry

The volatile compounds were desorbed from the Twister stir bar using an automated thermal desorption unit (TDU; Gerstel, Mülheim an der Ruhr, Germany) mounted on an Agilent 7890A GC system coupled to a quadrupole Agilent 5975C electron ionization mass spectrometric detector (MS; Agilent Technologies, Palo Alto, CA, USA) equipped with a fused silica capillary column (BP21 stationary phase 30 m length × 0.22 mm internal diameter × 0.25 µm film thickness; SGE, Ringwood, Australia). The carrier gas was helium with a constant column pressure of 20.75 psi. The volatile compounds were desorbed from the stir bar under the following conditions: oven temperature, 35 °C; desorption time, 3 min; cold trap temperature, −30 °C; and helium inlet flow, 1 mL/min. The chromatographic program was set at 35 °C (held for 5 min) and then raised to 150 °C by 5 °C/min and held for 1 min. For mass spectrometry analysis, electron impact mode (EI) at 70 eV was used. The mass range varied from 35 to 500 u, and the detector temperature was 250 °C.

Mass spectrometry data acquisition was performed in the positive scan mode; however, to avoid matrix interferences, MS quantification was performed in the SIM mode using their characteristic *m*/*z* values.

### 3.4. Analytical Method Validation

The method was validated according to the Eurachem guideline [24]. The following method performance characteristics were assessed: linearity, selectivity, detection and quantifications limits (LOD and LOQ), precision, and accuracy. For the linearity study, calibration graphs were generated by injecting standard solutions of saffron components (safranal, linalool, isophorone, and 2,6,6-trimethyl-2-cyclohexene-1,4-dione) at different concentrations. Cyclohexane and rice flour (10 mg) were used to obtain the starting solution. Each concentration was analyzed in duplicate, and two measurements were taken for each replicate. The selectivity was assessed using the majority ion of the corresponding mass spectra for quantification, comparing the chromatograms obtained from the analyzed solutions. The sensitivity was determined on the basis of the limit of detection (LOD) and limit of quantification (LOQ), considering the signal-to-noise ratio (S/N) for each standard. The LOD and LOQ concentrations were calculated by multiplying S/N by 3.3 and by 10, respectively. The accuracy of the method was evaluated on the basis of precision, taking into account the repeatability and the intermediate precision or reproducibility. The repeatability was determined using six different concentrations of each standard, performing 10 injections per concentration. Thus, the mean value of the peak areas obtained and the relative standard deviation (RSD) of each standard were calculated. The intermediate precision was determined by analyzing each standard at its intermediate concentration on three consecutive days by the same analyst. The mean concentration and the RSD value of the peak area were calculated for each standard. The repeatability and reproducibility were considered to be acceptable if the %RSD value was lower than 20%.

Finally, the applicability of the present method was determined by analyzing cured ham flavored with saffron. For this purpose, nine samples of saffron-flavored cured ham were purchased from the company Benibaldo, S.A.U.—three samples each of the three production lines in which different saffron-to-cured ham ratios are used. 

### 3.5. Statistical Analysis

One-way analysis of variance (ANOVA) was performed, and mean values were compared using Duncan’s test at *p* < 0.05 (95% confidence interval) using the statistical software package SPSS 24.0 (SPSS Inc., Chicago, IL, USA).

## 4. Conclusions

This study describes the development and validation of a new analytical method for determining the compounds responsible for saffron aroma in flavored cured ham. To our knowledge, this is the first study to identify and quantify the compounds safranal, linalool, isophorone, and 2,6,6-trimethyl-2-cyclohexene-1,4-dione from cured ham flavored with saffron. The analytical method was found to be adequate in terms of linearity, selectivity, sensitivity (LOD and LOQ), and accuracy. The proposed method can be used to evaluate the content of saffron volatile compounds in cured ham flavored with this spice, safranal being the main compound, and it could be of interest to agri-food industries. Furthermore, this analytical method could be used as a reference method to be adapted or to develop new ones capable of assessing the aromatic composition (its transfer) that the addition of other spices to cured ham would entail.

## Figures and Tables

**Table 1:** Linearity data for analysis by headspace-stir bar sorptive extraction-gas chromatography/mass spectrometry.

Standard	Linear Range (µg Standard/20 mL vial)	Regression Equation	R^2^
Safranal	0–5.109	y = (1.405 × 10^7^ ± 4.224 × 10^5^)x	0.999
Isophorone	0–5.982	y = (4.382 × 10^6^ ± 5.432 × 10^5^)x	0.991
Linalool	0–6.455	y = (1.560 × 10^6^ ± 1.344 × 10^5^)x	0.996
tMCHdO ^1^	0–4.517	y = (4.875 × 10^6^ ± 1.131 × 10^6^)x	0.985

^1^ tMCHdO: 2,6,6-trimethyl-2-cyclohexene-1,4-dione. R^2^: Correlation coefficient.

**Table 2:** Sensitivity and precision of the experimental procedure for analysis by headspace-stir bar sorptive extraction-gas chromatography/mass spectrometry.

Standard	LOD (µg Standard/20 mL vial)	LOQ (µg Standard/20 mL vial)	Repeatability (% RSD)	Reproducibility (% RSD)
Safranal	3.992 × 10^−4^	1.210 × 10^−3^	2.338	4.050
Isophorone	1.628 × 10^−3^	4.935 × 10^−3^	1.861	3.223
Linalool	2.590 × 10^−2^	7.847 × 10^−2^	2.200	3.811
tMCHdO ^1^	2.650 × 10^−3^	8.029 × 10^−3^	1.723	2.984

^1^ tMCHdO: 2,6,6-trimethyl-2-cyclohexene-1,4-dione. LOD: limit of detection; LOQ: limit of quantification. RSD: relative standard deviation.

## Sample Availability

Samples compounds (safranal, isophorone, linalool, and tMCHdO) are available from the authors.
